# Ecological networks to unravel the routes to horizontal transposon transfers

**DOI:** 10.1371/journal.pbio.2001536

**Published:** 2017-02-15

**Authors:** Samuel Venner, Vincent Miele, Christophe Terzian, Christian Biémont, Vincent Daubin, Cédric Feschotte, Dominique Pontier

**Affiliations:** 1 Laboratoire de Biométrie et Biologie Evolutive UMR5558-CNRS, Université de Lyon, Université Claude Bernard Lyon 1, Villeurbanne, Lyon, France; 2 LabEx ECOFECT (Eco-Evolutionary Dynamics of Infectious Diseases), Université Claude Bernard Lyon 1, Villeurbanne, Lyon, France; 3 UMR754 INRA, Université Claude Bernard Lyon 1, Lyon, France; 4 Ecole Pratique des Hautes Etudes, Paris, France; 5 Department of Human Genetics, University of Utah, School of Medicine, Salt Lake City, Utah, United States of America

## Abstract

Transposable elements (TEs) represent the single largest component of numerous eukaryotic genomes, and their activity and dispersal constitute an important force fostering evolutionary innovation. The horizontal transfer of TEs (HTT) between eukaryotic species is a common and widespread phenomenon that has had a profound impact on TE dynamics and, consequently, on the evolutionary trajectory of many species' lineages. However, the mechanisms promoting HTT remain largely unknown. In this article, we argue that network theory combined with functional ecology provides a robust conceptual framework and tools to delineate how complex interactions between diverse organisms may act in synergy to promote HTTs.

## Introduction

Horizontal DNA transfer, or the passage of genetic material between organisms by means other than reproduction, while commonly observed in bacteria [1,2], has long been considered rare between multicellular eukaryotic species, with negligible impact on their evolution [3]. However, an increasing number of recent studies, in part fueled by the exponential growth of genome sequencing, have revealed that the transfer of genetic material between multicellular eukaryotes has occurred more commonly than previously appreciated (e.g., [4–9]; for recent reviews, see [2,10,11]). Among the well-documented cases of horizontal transfer between multicellular eukaryotes, those involving transposable elements (TEs) are by far the most common (for reviews, [5,12,13]). The propensity for TEs relative to non-TE sequences to undergo horizontal transfer may in part be attributed to their inherent mobility [5,14] and to their capacity for rapid genomic amplification following their introduction; this would facilitate the spread of these elements in populations even in the absence of an immediate fitness advantage to the host [15]. In fact, the horizontal transfer of TEs (hereafter “HTT”) can be viewed as a crucial process for the maintenance and propagation of TEs in eukaryotic genomes [5,16]. Consistent with this view, examples of HTT have rapidly accumulated in the literature over the past decade. Notably, a considerable number of HTT events have been reported among *Drosophila* species, in part because *Drosophila* represents a prominent model in evolutionary genetics [17] and also because the large number of genomic data available makes them particularly amenable to the development and application of robust statistical approaches to detect HTTs [18]. However, recent studies have uncovered solid cases of HTTs in an increasingly wide range of eukaryotic species as reviewed previously [5,12,13] and illustrated more recently by a flurry of new cases involving a variety of invertebrates, vertebrates, plants, and some of their parasites [19–25]. Hence, a wide range of species and all major types of TEs are known to be implicated, regardless of the diversity of their structures and transposition mechanisms [5,12,13,24,25]. Since TEs represent a major component of the nuclear genome of multicellular eukaryotes and an important source of genetic variation catalyzing evolutionary innovation, HTT should be regarded as a pivotal force in eukaryotic genome evolution (Box 1).

**Box 1. HTT as a fundamental step in the life cycle of transposable elements**

Transposable elements (TEs) represent the single largest component of many large eukaryotic genomes, accounting for at least half of the human genome and an even greater fraction of other complex genomes [26–28]. While these elements are best described as genomic parasites [29,30], their accumulation and movement are now recognized as a prolific source of mutation and genetic rearrangements, greatly influencing the evolutionary trajectory of their host species and organismal evolution (for reviews, see [14,26,31–35]). As nearly ubiquitous components of eukaryotic chromosomes, TEs are transmitted vertically, i.e., from parents to offspring. However, it has long been appreciated that their persistence over vast evolutionary eons implies an ability to cross species boundaries and invade new genomes through horizontal transmission [5,16]. Following a single horizontal transfer event, a TE copy may rapidly spread through the recipient host population by means of high transposition activity combined with vertical transmission [36]. This initial expanding phase in a new population seems to be crucial because it sets the number of TE copies and their location in the host genome; this provides the breeding ground for future genetic alterations that can impact the evolutionary trajectory of the recipient TE-host species. Constantly high TE transposition activity and/or an ever-increasing abundance of active TE copies in the recipient species is expected, however, to result in excessive genomic instability, which ultimately will be incompatible with the survival of the host individual [15]. Consequently, TE activity is silenced by a variety of host-encoded strategies (such as RNA interference and other small RNA-based mechanisms, DNA methylation, histone modifications, and chromatin changes) as well as self-regulatory mechanisms [37–39]. In the face of these mechanisms, empirical and theoretical studies have shown that, in the absence of natural selection acting at the host level to maintain transposition activity, the frequency of active TE copies is bound to decrease in the population and that of defective copies to increase, eventually leading to the extinction of the entire TE family [15,40,41]. Horizontal transfer represents one mechanism by which TEs can escape such extinction by providing an opportunity to colonize new host genomes and repeat the cycle [5,16].

TEs are classified into different families according to their transposition mode or genetic structure (see glossary), and some of them seem to have greater aptitude for HTT than others [42]. Some long-terminal-repeat (LTR) retrotransposons (e.g., the *gypsy* element in Drosophila), like retroviruses, are capable of producing a functional envelope protein [43,44] that gives them an intrinsic ability to infect new cells [44]. Nevertheless, HTTs have been shown to occur for all major TE types, irrespective of their ability to encode envelope proteins [5,13].

The biological factors and cellular mechanisms promoting HTT in eukaryotes remain poorly understood. There is a growing body of evidence pointing at the role of parasites and pathogens (e.g., viruses, bacteria, or macroparasites [ecto- or endoparasites]) in facilitating HTT [17,22,24,45–48]. Yet, to our knowledge, no published attempt has been made to provide a robust framework to synthesize and integrate genomic and ecological data in order to illuminate how complex biological interactions between organisms may promote HTT. Here, we argue that network theory is a powerful approach to characterize the dynamics and disentangle the forces underlying HTT. Network theory delivers a set of tools to effectively model complex systems (i.e., composed of interacting entities) and to analyze their emergent properties, as utilized in physics, social sciences, ecology, and, more recently, in cell biology [49–51]. In the area of genomics, we argue that complex systems formed by organisms having complementary properties and working synergistically to support HTT can be formalized using ecological networks, which represent complex interactions between organisms within ecological communities. The emergent property of the network is the shared presence of TE acquired through HTT in otherwise unrelated eukaryotic genomes. Network analysis would thus allow deciphering which organisms and which of their interactions are prone to promote HTT and thereby play a key role in the evolutionary dynamics and maintenance of TEs.

## Current approaches to study HTT

Successful HTTs between multicellular and sexual eukaryotic species generally require that (1) one copy of a TE from a donor species reaches the germ line of an individual of the recipient species and is integrated into its genome, (2) germ cells integrating new TE copies produce fully functional gametes, and (3) TE spreads within the population of the recipient species through further transposition into the host genome and vertical transmission of newly formed copies. The successful fixation of the TE in the novel host genome site depends on both genetic drift and selective processes [52]. Note that a TE need not be fixed in the population to reveal HTT. In fact, polymorphic TE insertions are likely to indicate more recent HTT and hence be most relevant for illuminating extent ecological links. Intuitively, the probability for all these steps to be achieved must be extremely low, yet unequivocal cases of HTTs have now been well documented (see references above). The participation of intermediate biological vectors is often evoked to explain HTT across widely diverged species, but we note that the direct transfer of nucleic acids or nucleoprotein complexes, either as free molecules or packaged in extracellular vesicles, is also conceivable (see Box 1) [5,13].

The great majority of studies investigating HTT have been “species-centric”, focusing either on the identification of organisms that carry TEs or on the type of species interactions promoting HTTs. Many investigations have concentrated on parasitic or symbiotic microorganisms (viruses, bacteria, endosymbiotic bacteria, and unicellular eukaryotes) that are most commonly evoked as TE vectors because of their established propensity to transduce and recombine genetic material from their host [7,43–51]. Among these potential TE vectors, viruses appear particularly suitable because of their defining ability to enter and exit cells, their propensity to capture and deliver genetic material from and to their host genome, and their capacity to infect germ cell lineages or their precursors [43,52–54]. Similarly, bacteriophages are considered a major source of gene transfer in bacteria [2,53]. Large double-stranded DNA viruses represent the most outstanding candidates for facilitating HTTs among eukaryotes, as suggested by numerous reports of TEs clearly derived from a eukaryotic host integrated in their genomes [5,12,13,42,46,54–60]. RNA viruses might also promote HTT when TE RNAs are encapsidated and copackaged along with viral genomic RNA [61]. Likewise, “virus-like particles” created by long-terminal-repeat (LTR) retrotransposons and endogenous retroviruses, which are well characterized in vertebrates and have been also detected in insects [62], can enter recipient cells and be transmitted to other organisms (see S1 Text for an expanded discussion).

Other investigations have aimed at identifying the routes for HTT among phylogenetically distant species by building comparative analyses of genomic composition of TEs across species. These studies have implicated macroparasites (e.g., flatworms, filarial nematodes, strepsipteran insects, and blood-feeding triatomine bugs, ticks, and lampreys) as facilitating the passage of various TEs between distantly related hosts [20,22,45,47,59,63–65], but predator–prey interactions may also establish a route for HTT [20].

Taken together, these studies suggest that HTT is promoted by various ecological interactions between a wide diversity of organisms. Faced with this complexity and the ever-growing amount of genome sequence data for a wide range of organisms, it has become a necessity to develop a conceptual framework to disentangle the relative importance of the factors and processes underlying HTT. In complement to ecological theories of biodiversity already considered in previous studies (see [66] for a review), we propose that network theory combined with functional ecology provides an adequate conceptual framework and a toolbox to formalize and analyze, from large datasets, the multiplicity of mechanisms and routes underlying HTTs.

## A functional ecology perspective on HTT

Functional ecology concentrates on the functional roles of species in the community by focusing on their traits and by analyzing their impact on community dynamics or ecosystem processes. Using networks in a functional ecology approach to understand HTT requires defining the functions necessary to ensure HTT and identifying different classes of organisms that might fulfill these complementary functions (based on their traits) and act synergistically.

### Which requirements for HTT?

**Fig 1:**
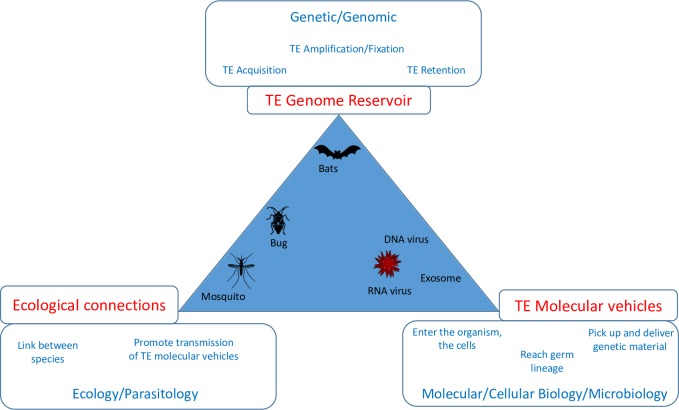
Requirements for HTTs. The figure presents the three complementary functions (defined in the main text) expected to modulate HTTs. The frames near the vertices of the triangle specify the properties required for organisms to ensure those functions and indicate the subdisciplines of biology for identifying them. The triangle allows viewing different gradients along which could be positioned different organisms involved in HTTs. Some eukaryotic species, like bats, would be particularly good TE reservoirs. Other species, like triatomine bugs, would be efficient both in the role of TE reservoir and as ecological connectors and might consequently operate as large hubs in TE dynamics. Other organisms, like DNA or RNA viruses, seem to have the necessary requirements for being efficient “TE molecular vehicles.” Poxviruses, in some circumstances, seem able to play the three functions, alone ensuring the HTT between ecologically close eukaryotic species.

We identify three complementary functions that must be fulfilled for successful HTT (Fig 1):

1. “Molecular vehicle function” reflects the ability to capture TEs in the genome of a donor species and transmit them to the genome of a recipient species. Different types of viruses and intracellular microparasites of eukaryotic cells exhibit highly suitable characteristics to act as molecular vehicles (see S1 Text).
2. “Reservoir function” corresponds to the acquisition and storage of TE copies within a given population or species over evolutionary time; this function would determine how long a species might act as a “launching platform” for new HTT events. High reservoir ability should be associated with high rate of TE acquisition (e.g., low capacity of the immune system to control the entry and replication of molecular vehicles and/or large population size [67]) and high TE proliferation/maintenance within the genome (e.g., large genome size, vast areas of “dispensable” DNA, and/or slow rates of substitution and deletion, which could help to preserve its intact copies). For example, mammalian genomes exhibit many of these characteristics.
3. “Ecological connection function” represents the ecological link between different eukaryotic species. The intensity of each link and its direction reflect the frequency of interactions between individuals of the two species, the frequency and direction of transfers of “TE molecular vehicles” during those interactions. Recent studies suggest that host–parasite and prey–predator interactions are the most probable ecological connections involved in HTT [20,45,47,59].

### Synergy between organisms promoting HTT

A wide variety of organisms (micro- and macro-organisms) may be involved in HTT. It is, however, highly unlikely that any single organism could efficiently fulfill the three functions defined above. On the one hand, intracellular microparasites such as viruses constitute excellent molecular vehicles for TEs (see S1 Text). However, TEs acquired by viruses from their host, like other nonessential DNA, may be expected to be rapidly removed from viral genomes owing to their fitness cost and the large effective population size of viruses [68]. Consequently, while viruses are likely involved in HTTs among diverse species (e.g., poxviruses, which are known to have a broad host range or to switch host frequently [56]), they seem inadequate to act as long-term TE reservoirs [60], that is, to “store” TEs in their genome for a long enough time to promote their transfer on a wide scale—i.e., between multiple, phylogenetically distant eukaryotic species. On the other hand, some macro-organisms (e.g., those with large genomes, slow mutation rates, and small effective population size, such as vertebrates) are good candidates to store active TEs over a long period and thus act as TE reservoirs. Furthermore, the ecological relationships between macro-organisms (between generalist macroparasites and their hosts or between generalist predators and their prey) can create links to establish a route for HTTs. However, macro-organisms alone (e.g., macroparasites, hosts, prey, or predators) would only be capable of delivering nonreplicative transposition intermediates (i.e., nucleoprotein complexes, partially degraded DNA, or DNA encapsulated in vesicles of cellular origins, such as exosomes) into the circulating fluid (e.g., blood, hemolymph, and sap) of a recipient species (their hosts, macroparasites, predators, or prey, respectively). Such intermediates are not typically self-replicative and therefore are unlikely to reach and infiltrate the germline of the recipient species by themselves. Consequently, macro-organisms alone may not be sufficient for successful HTT. Altogether, these limitations suggest that the most optimal path for HTT might require a complex interplay between very diverse organisms (e.g., both micro- and macro-organisms) acting synergistically to facilitate the process.

An example illustrating the intermingled action of a virus and a macroparasite in HTT is given by the peculiar case of the tripartite system composed of parasitoid braconid wasps, their symbiotic polydnaviruses, and their lepidopteran hosts. Polydnaviruses are integrated as proviruses in the wasp genome and produce viral particles in wasp ovaries that are injected into the lepidopteran host at the same time as wasp eggs, allowing wasp larvae to evade the immune response of their lepidopteran hosts [69]. Sequences representing diverse TEs (*mariner*-like, *gypsy*-like, *Maverick*-like, and *DIRS*-like elements) were detected in the genomes of polydnaviruses [54,70], suggesting that TEs originating from the wasp genome may be frequently copackaged and delivered to the lepidopteran cells via the virus-like particles produced by polydnaviruses [61,71].

More generally, vector-borne viruses (or microparasites) and their biological vectors could constitute ideal pairs of organisms to connect genomes of diversified host eukaryotic species. Here, the viruses, their intermediate vectors (e.g., ecto- or endomacroparasites), and their final host species would act in synergy by playing the respective functions of molecular vehicles, ecological connectors, and reservoirs. In a context where the viruses multiply within their vector and where the virus vector is in contact with numerous and diverse host species during its evolutionary trajectory (e.g., generalist), the virus vectors can simultaneously act as TE reservoirs and ecological connectors linking a wide range of eukaryotic species. Such macroparasites might consequently operate as large hubs for HTT. Such species may include blood-feeding parasites such as triatomine bugs, ticks, or lampreys, which are also common vectors of microparasites and have been found to share TEs nearly identical to those of some of their known vertebrate hosts [45,47,59]. Similarly, predator–prey relationships, combined with virus transmission, could play an important role in HTT. Like generalist vectors, generalist predators could accumulate TEs of various origins according to the diversity of consumed prey and to their capacity for infection and replication of viruses acquired from prey [58,72–75].

In sum, we envision the process of HTT as supported by a complex system of interacting organisms, which act in synergy at many different time and spatial scales ranging from cellular processes to community dynamics. Next, we argue that network theory offers a powerful way of representing and characterizing such a complex system to better comprehend the process of HTT.

## Network theory to unravel HTT

Fundamentally, a network provides a framework to model the pairwise links among a set of objects having contrasted properties and to explore the emergent properties at the scale of the whole system. We detail below how networks can be used to model HTTs (hereafter “HTT networks”), and we illustrate, from simulation, their emergent properties, i.e., the similarity of genomes in TE composition. Then, we identify near-term prospects for the construction of HTT networks from empirical data to improve our understanding of the dynamics of TE movements between eukaryotic species.

### HTT network characteristics

Using network approach in the context of HTT involves defining three characteristics: (1) the network topology, which captures the diversity of organisms potentially involved in HTT as well as their functional roles and their links; (2) the flow within the network, i.e., the dynamic of TE propagation among the species, which is based on their synergistic action; and (3) the network's emergent properties (or the network state resulting from that dynamic process), which correspond here to the degree of similarity of genomes of distinct species in terms of TE composition, resulting from HTT.

#### Topology of HTT networks and properties of their elements

An HTT network can be represented as a set of nodes, each node corresponding to a TE reservoir, and edges, which map the connectivity between the nodes. Both nodes and edges can have multiple characteristics and might not be all weighted equally. Nodes can represent eukaryotic species or potentially prokaryote species as soon as they have abilities to maintain TEs in their genome for sufficient time to give future opportunities of HTT. Each node is characterized by its reservoir ability, i.e., the ability to maintain/amplify TEs, which is taken into account in our basic model by a parameter indicative of the maximal number of TE copies that the genome can carry (see S2 Text). The node connectivity depends on the number of pairwise links with other nodes and on the strength and direction of each link and therefore depends on the ecological relationships among reservoir organisms. These relationships can be asymmetric (e.g., a predator eats a prey) or symmetric (e.g., a virus infecting multiple host species). Furthermore, the strength of a link represents the density of “TE molecular vehicles” and the facility with which they transit along the link, capture, and deliver TEs. Finally, multiple types of HTT networks can be built to represent the variety of ecological contexts that can be encountered (see Box 2 for details).

**Box 2. Various topologies for HTT networks**

Network topology, which reflects species relationships and captures the properties of nodes and links, provides the foundation for modeling HTT. Networks with contrasting topologies can be built (see S1 Fig) [76]. Although they are presented separately here, they can be partially combined according to ecological contexts, to form more complex networks.

**Fully connected (or complete) networks**

Within these networks, all nodes are directly interconnected (i.e., any possible edge is present). In the context of HTT, this implies that the movement of TEs is not constrained by any particular ecological relationship (i.e., all species interact with each other in the same way). This type of network, although not realistic, reflects optimal conditions for TE spread and can be used to evaluate how ecological interactions impede and channel TE flows.

**Random (or Erdös-Rényi) networks**

Within these networks, the basic connectivity (number of links per node) follows a Poisson distribution. Node connectivities that strongly deviate from the average connectivity are extremely rare, and consequently, there are typically no hubs, i.e., nodes with very high connectivity relative to other ones. Such networks would be considered as null/neutral HTT networks reflecting no ecological structure.

**Scale-free networks**

The node connectivity follows a power-law distribution that is characterized by a relatively small number of highly connected nodes corresponding to hubs. A similar pattern may also be obtained from exponential connectivity distribution [77]. Such networks may reflect situations in which HTT is ensured by viruses that would play the dual role of ecological connectors between eukaryotes and TE molecular vehicles (e.g., airborne viruses). Hubs would correspond to eukaryotic species with a great capacity of “TE reservoir” particularly exposed to viruses coming from numerous species. These hubs would therefore play the major role in centralizing and disseminating TEs. For instance, some bats might act as a hub—in particular, the species that (1) can air-travel great distances and are in contact with multiple species (bats of various species prey heavily on insects that transmit viruses, while others eat vertebrates like frogs, rodents, birds, fish, or other bats or feed on the blood of other vertebrates), (2) harbor large loads of a variety of viruses [78] which are potentially molecular vehicles of TEs, (3) have a high TE content in their genome and thus a capacity to be a TE reservoir [79], and (4) share TEs with many other eukaryotic species [20,45,57,72,75].

**Modular networks**

These networks are composed by groups of nodes (“modules”) that have more connections within than between groups. These networks capture ecological interactions characterized by a partial isolation of species groups, with species strongly interconnected within each group. Such networks could account for spatial isolation of groups of species that evolve on different continents and between which occasional exchanges may occur (e.g., via migratory animals and their macroparasites/viruses) [22,45]. Modular networks might also be useful to model partial isolation between ecosystems and communities (e.g., marine and terrestrial communities/ecosystems). Indeed, a recent study [23] suggests that the horizontal spread of TEs is more likely to occur between aquatic species.

**Bipartite networks**

These networks model asymmetry of interactions between nodes and are often used in ecology in the context of plant–pollinator, prey–predator, or host–parasite interactions. They are defined by two disjoint sets of nodes with direct interactions between, but not within, sets. Such bipartite networks would capture the synergistic actions of the different organisms potentially involved in HTT. For example, one set of nodes may correspond to a macroparasite species, another one to their potential hosts, and the link between sets would be established by vector-borne viruses. Macroparasites may include blood-feeding triatomine bugs, ticks, or lampreys, which are common vectors of microparasites and have been found to share TEs nearly identical to those found in their vertebrate hosts [45,47,59].

#### Dynamic of TE flows within the HTT networks

Once defined, the network topology provides the foundation for modeling TE flows between nodes (i.e., organisms’ reservoirs of TEs). This step requires taking into account both the intragenomic dynamics of TEs (amplification dynamics and persistence of TEs within genomes, which constitute the source for further HTT) within each node and the dynamics of transfer along the network links. To explore the interplay between network topology and TE dynamics, we developed a basic model (detailed in S2 Text) based on probabilistic simulations of flows of different TE families in a network of eukaryotic species. The simulations provide some avenues for HTT network analysis.

#### Emergent properties of networks: Similarities in genomic TE composition

**Fig 2:**
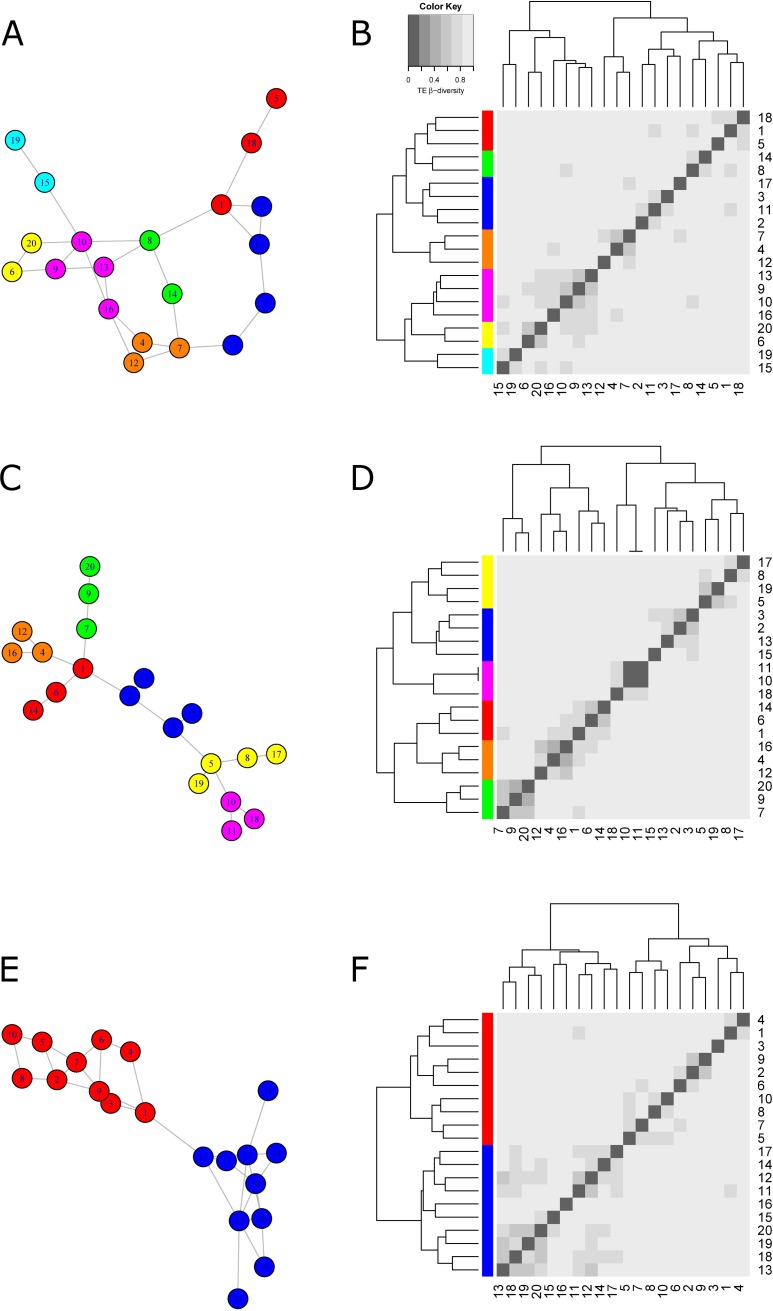
The simulated *β*-matrix representative of the HTT network. Panels A, C, and E represent a random, a scale-free, and a modular HTT network, respectively; panels B, D, and F represent the corresponding simulated *β*-matrices obtained after simulation of TE dynamics along the given random HTT network, with 20 species, 30 TE families, and 150 HTTs. Species (numbered 1 to 20) are ordered with a hierarchical clustering based on TE *β*-diversity. The heatmap scale is indicated from the grey gradient shown in panel B. The simulated *β*-matrices exhibit blocks of species of similar TE content (panels B, D, and F) that can be retrieved by an appropriate cut of the dendrogram (different colors are used for the leaves of the different subtrees induced by this cut). Interestingly, these blocks are topologically coherent in the HTT network (panels A, C, and E). Parameters of the model are given in S2 Text. Networks were represented with the R igraph package with the "nicely" layout.

We explore the emergent property of TE flows from simulations. The simulation results can be synthetized by building a matrix crossing all pairs of genomes (network nodes), in which we indicate the similarity in TE composition between each pair of genomes/nodes based on the presence/absence of TE families within those genomes/nodes (Fig 2). The emergent property of TE flows within a network is therefore the expected degree of similarity between genomes (nodes) in terms of shared TEs acquired from horizontal transfers. Here, we choose the Jaccard similarity coefficient [80], which is equivalent to *β*-diversity, an index widely used in community ecology that was introduced by Whittaker [81], to measure the degree to which species composition differs (from the presence/absence of species) between communities, i.e., between different localities of a same region. By analogy, the “TE *β*-diversity” would quantify the degree of differentiation in TE composition between a pair of species belonging to the HTT network and thus will be used to build the “simulated TE *β*-diversity matrix,” hereafter called “simulated *β*-matrix” (the modeling procedure of TE flows implying several families are detailed in S2 Text).

#### The *β*-Matrix, a powerful tool for discriminating HTT networks

**Fig 3:**
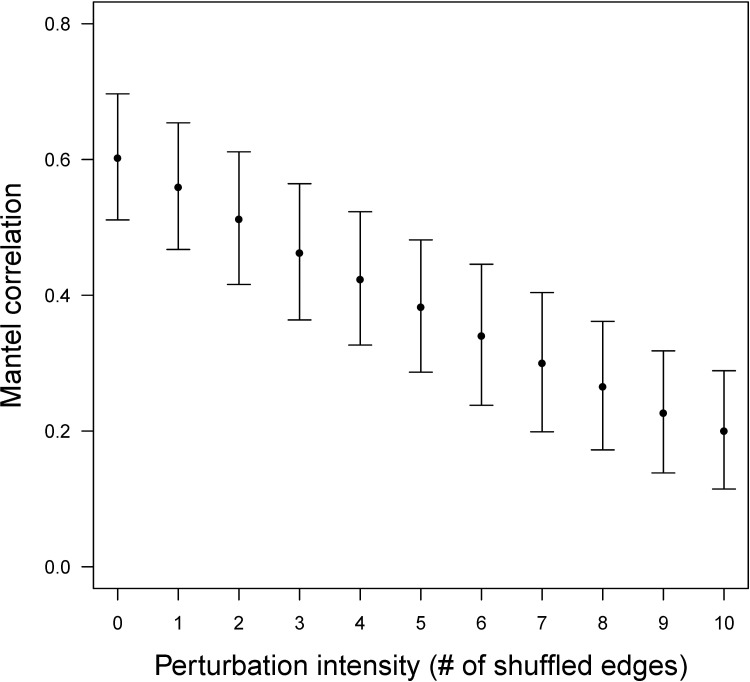
The simulated *β*-matrix as an efficient tool to discriminate among HTT networks. Distribution of Mantel correlation coefficient (mean +/− standard deviation [SD]) between a reference *β*-matrix obtained for a given HTT network and a *β*-matrix obtained on a disturbed HTT network (perturbation is expressed by the number of edge shuffles [*x*-axis] in the given HTT network; no shuffled edge indicates that the network is unchanged). We used the same simulation settings as in Fig 2. The procedure was repeated for 50 random scale-free HTT networks and 10 replicates of each number of edge shuffles. Similar results were obtained by reducing the HTT rate to transposition rate ratio (see S1 Fig). The pattern is similar when using lower numbers of successful HTT; however, the level of correlation increases with the number of HTTs within the network (see S2 Fig).

We show that the *β*-matrix constitutes a powerful tool for characterizing and discriminating HTT networks. First, using a characteristic case based on a random sparse, scale-free, and modular network (see Box 2), we show that the simulated *β*-matrices should allow the groups of connected species as defined by the structure of the original network to be recovered (Fig 2). Second, we performed a systematic simulation analysis in which we show that simulated *β*-matrices are stable for a given HTT network (Fig 3 shows a strong correlation between simulated *β*-matrices when there is no shuffled edge in the HTT network, i.e., when this network remains unchanged). Conversely, an increasing level of perturbation in network connectivity (i.e., an increasing number of shuffled edges in the network of reference) leads to a decreased correlation between simulated *β*-matrices (Fig 3). Different HTT networks give rise to different *β*-matrices, which provide powerful tools to discriminate even among HTT networks close in their topology. Together, these results strongly suggest that it will be possible to reconstruct the topology of a HTT network from genomic data collected in the different species included in the network.

### Perspectives to promote the development of HTT network approaches

#### Reconstruction of an HTT network from genomic data

Reconstructing an HTT network from genomic data should allow the identification of key components, species, and/or links characterizing the propagation dynamics of TEs. The determination of the “empirical *β*-matrix” will be the first step for reconstruction of one (or more) plausible HTT networks. As for the simulated TE *β*-diversity, the empirical TE *β*-diversity between pairs of species will be calculated from the presence/absence of various TE families. Typically, this can be done based on genome sequences acquired from next-generation sequencing approaches. Because shared TE families can arise from vertical descent, especially when closely related species are under consideration, one will need to choose a similarity criterion for the grouping of TEs in a family that accounts for the expected divergence among the genomes considered [5,12]. In general, more stringent similarity criteria will reveal more recent HTTs and hence be more readily interpretable in terms of the extant network. Relaxing the similarity criterion for detecting HTTs bears the risk of including more ancient links or links to unknown related species rather than those under consideration and hence would introduce spurious links in the network.

Once the empirical *β*-matrix is determined, the reconstruction of the topology of the corresponding most likely HTT networks would greatly benefit from the many methodological advances in other disciplines (e.g., physics, social science, neurobiology, and community ecology) (see Box 3 for details). One major difficulty of the HTT network reconstruction can be related to the fact that some key species are missing (e.g., species not sampled or extinct). We note, however, that specific methods exist to detect hidden nodes [82]. In our context, the hidden nodes would correspond to reservoir organisms whose incorporation in the network topology would help to recover the empirical *β*-matrix. Their detection through the network analysis would provide a useful guide for future effort to uncover novel players (e.g., virus and bacterium) with a major role in HTTs.

**Box 3. Network reconstruction methods**

Knowing the presence/absence of each TE family and the empirical *β*-matrix, the challenge remains intact regarding the reconstruction (or inference) of the HTT network. Meanwhile, there is room for new methodological developments that could take their inspiration from methods developed in other domains. Indeed, this “reverse-engineering” problem could benefit from the cross-fertilization of ideas developed to reconstruct different kinds of networks (neuroscience with brain networks [85], bioinformatics with gene regulatory networks [86], ecology with food webs [87], or network science [88]). We propose here to pave the way for future developments by mentioning some ideas:

- Simple measures of correlation between nodes are often used to infer an initial version of a network, which can be completed or modified by expert knowledge. Here, since any element of the *β*-matrix contains a measure of *β*-diversity in TE, the matrix can be used as it is to infer putative edges (see [89] for a discussion on this approach for social networks and [90] for gene networks).
- Numerous available methods are based on mutual information (MI). The MI measures the amount of information that one node contains about another—in other words, the point to which the TE contents of two nodes are redundant. Some implementations are available (e.g., R package Minet [91]; see also [90]) and could help in the first attempt at HTT network reconstruction.
- Simulation-based methods can also face the challenge of deciphering the original HTT network among the huge quantity of possible networks. Indeed, approximate Bayesian computation methods (ABC [92]) can be applied in our context: it consists in simulating the TE dynamics—as presented here—on numerous candidate networks (possibly preselected by the previous methods, in order to restrict the space of possibility) and selecting the candidate for which the TE *β*-matrix is the closest to the original one (in the sense of the Mantel correlation).
- Other popular methods based on probabilistic graphical models (for instance, the Markov random field, but not Bayesian networks, since HTT networks are not directed) could be adapted to our problem, as well as penalization techniques that allow for sparse reconstruction [93].
- The HTT matrix reconstruction problem could also be formulated into a combinatorial optimization problem: knowing the presence/absence of each TE family in the genomes, the reconstruction eventually ends up as a multiweighted Steiner tree problem [94]; here, there is one weight function per TE family.

As perspective, the performance of the different methods could be evaluated by comparing their ability to reconstruct HTT networks, for example, according to the number of species, the number of TE families, the number of HTT events in the network, or whether or not known ecological relationships between species are introduced.

Once the network topology has been reconstructed (qualitative characterization of the connectivity between nodes), it will then be possible to weight the different components of the network (quantitative characterization of the network elements, i.e., the reservoir capacity of the different nodes and the intensity of the links) in order to maximize the efficiency of the HTT network to recover the similarity of the genomes in their TE composition. Network reconstruction drawn from the quantitative and qualitative approaches would permit major interactions and keystone species—i.e., those that are expected to largely impact the dynamics of HTTs because of their expected high TE reservoir abilities and/or because they constitute hubs and are thus expected to build many links with other species—to be pinpointed. The network should then stimulate further research to identify the peculiar properties of those species and their links (e.g., study of their virome, molecular, cellular, and physiological properties, and their ecological interactions).

#### HTT networks link ecology and genomic properties

In addition, HTT networks would provide powerful tools for testing formally the relative importance of certain identified ecological interactions, ecological isolation, or the involvement of certain categories of molecular vehicles (see S1 Text) in the dynamics of HTTs.

The reconstruction process can indeed introduce some a priori structural constraints, which are derived from ecological knowledge and capture key structural properties of the most complex and comprehensive food webs [83,84]. To illustrate simple cases, we can impose a bipartite network structure to capture host–parasite or prey–predator interactions (with potential constraints on the direction of HTT along links) or a modular network to capture geographically isolated species or groups of species living in ecosystems that are partially isolated (see Box 2). We illustrate that different topological networks can generate different dynamics of TE propagation (see S3 Fig), which as a result would ultimately affect the distribution of TE families in the genomes. It will then be possible to assess the ability of such networks to recover similarity or divergence of genomes in their TE composition and thus to infer the relative importance of ecological interactions on TE dynamics.

As a complementary example, network analysis could also be used to identify which molecular vehicles, among all potential molecular vehicles, could play a key role in HTTs (e.g., RNA versus DNA viruses; see S1 Text). Such an approach would consist in reconstructing the networks that underlie the transfer of molecular vehicles (by building the “molecular vehicle *β*-diversity matrix” from the similarity of species of the network in their composition in those vehicles) and testing the capacity of these networks to generate the similarity of genomes in their TE composition.

In summary, while the application of network theory in the context of HTT will require substantive methodological developments, the approach is bound to deliver powerful tools to unravel the complex mechanisms governing the dynamics of propagation of TEs in eukaryotic species.

## Conclusion

Here we argue that the conceptual framework and methodological tools provided by network theory can shed new light on the process of HTT. Applying this approach is becoming increasingly feasible thanks to the affordability of genome sequencing and the exponential accumulation of genome sequence data for a wide range of organisms (outside of model species). This outpouring of genome sequence data, together with new analytical tools to systematically detect HTT [12,18,95], should soon enable the placement of a large number of HTT events across a dense network of species, as well as the assembly of an empirical HTT matrix. The increasing availability of public databases on relationships between eukaryotic species as well as their relationships with their microbial communities (e.g., DNA-based diet analysis, host–vector relationship, virome, and microbiome) will allow further exploration of the role of these factors in shaping the HTT network and ultimately the evolutionary dynamics of TEs.

The application of network theory in ecology has yielded profound new insights into the dynamics of communities and ecosystem processes from the properties of interacting organisms [96,97]. We argue that this approach can be adapted to provide a new conceptual framework and methodology to unravel the dynamics of TE movements between eukaryotic species, with TEs being virtually ubiquitous throughout the tree of life. The development of HTT networks will promote cross-disciplinary insights and the merging of concepts and knowledge borrowed from a vast array of biological areas, including ecology, genetics, genomics, cell biology, virology, bacteriology, and parasitology. Such an integrative approach will open up new avenues to perform and interpret large-scale analyses of genome composition resulting from HTT and, consequently, to better understand a pivotal process in the evolution of multicellular eukaryotes.

## Supporting information

S1 Fig Simulated β-matrix: Effect of the ratio between HTT rate and transposition rate on the distribution of Mantel correlation coefficient. Same legend as in Fig 3 (main text). We used the same simulation settings as in Fig 3 except for the ratio between the HTT rate and the within-genome transposition, which is one per 1000 (panel A) *versus* one per 100 (this figure, panel B and Fig 3). The result is insensitive to that ratio because (i) the intra-genomic dynamic of TEs is always much faster than their inter-genomic dynamic and (ii) the criterion used to end the simulation remains the number of successful HTTs (here it equals 150).
(PDF)

S2 Fig Simulated β-matrix: Effect of the number of successful HTTs on the distribution of Mantel correlation coefficient. Same legend as in Fig 3 (main text). We used the same simulation settings as in Fig 3. We tested the effect of the number of HTTs on the distribution of Mantel correlation coefficient (n = 50, 100, and 150 for panels A, B, and C, respectively). We show similar trends for all the tested situations (an increasing level of perturbation in network connectivity leads to a decreased correlation between simulated *β*-matrices). However, the level of correlation increases with the number of successful HTTs within the network. This result means that the network reconstruction will be all the easier when the number of HTTs is high within the species group considered.
(PDF)

S3 Fig Dynamics of propagation of a single TE family within contrasted networks. Panel A shows different structures of networks in which the nodes (species) have the same average degree of connectivity: random (A1), scale-free (A2), bipartite (A3), and modular (A4) networks (see Box 3 for a detailed description). Panel C represents random networks with different link densities, with the extreme case corresponding to the complete network. Panels B and D describe the diversity of dynamic of propagation of a single TE family in the network. The x-axis represents the number of species infected by the TE. The y-axis corresponds to time (number of iterations) needed to contamination of x species (x-axis) in the network. The box-plots represent the distribution of the time required for the contamination of x species. The width of the box-plot represents the proportion of trajectories in which the TE has contaminated x species. The figure shows networks with distinct topologies (panel A and C) and the dynamics of spread of a unique family of TE in these networks (panel B and D). For each category of networks (panel A and C), 400 simulations were performed. At the beginning of each simulation, a single copy of TE is placed in one of the species of the network, all the 20 network species being tested (20 different initial conditions/modality were therefore tested; 20 repetitions were performed per modality). Panels A and B show that the network structure affects the distribution of the TE propagation dynamics. The scale-free and modular networks generate dynamics different from those obtained in the case of random networks. The scale-free networks generate a very large diversity of TE propagation speeds: TE can spread either very rapidly when hubs are quickly contaminated or very slowly when hubs are slow to be contaminated. In modular networks, trajectories leading to the contamination of a large number of species are rare (strong decrease in the proportion of trajectories with more than 12 contaminated species) because of the presence of partially isolated groups of species. The dynamics of TE propagation in bipartite networks (*e*.*g*., host-macroparasite type) seem very close to those obtained from random networks, and therefore further analyses will be needed to detect the impact of bipartite networks on TE propagation. For example, the similarity in TE composition between very distantly related species (*e*.*g*., bug and their hosts) in a bipartite network is expected to be much greater than the similarity expected between those species in a random network. Panels C and D display the dynamics of TE propagation in different types of random networks differing by their average degree of connectivity (equal to 2, 3, 4, and the complete network having a maximum link density). The results (Panel D) show that the increase of link density greatly increases the speed of TE propagation and reduces the variability of propagation speed between the simulated TE trajectories. All these results emphasize the importance of ecological network structure in the dynamics of propagation of a single TE family, which should significantly alter the TE composition/similarity of genomes.
(PDF)

S1 Text Which molecular vehicles for HTT? (DOCX)

S2 Text Methods for modeling TE dynamic within the HTT network. (DOCX)

## Abbreviations

ABC: approximate Bayesian computation methods
HTT: horizontal transfer of transposable elements
LTR: long terminal repeat
MI: mutual information
SD: standard deviation
TE: transposable element
