# Supplementary figures and images for: Ecological networks to unravel the routes to horizontal transposon transfers
Source: PLoS Biol. 2017 Feb 15;15(2):e2001536. doi: 10.1371/journal.pbio.2001536 (PMC5331948; doi:10.1371/journal.pbio.2001536)

A

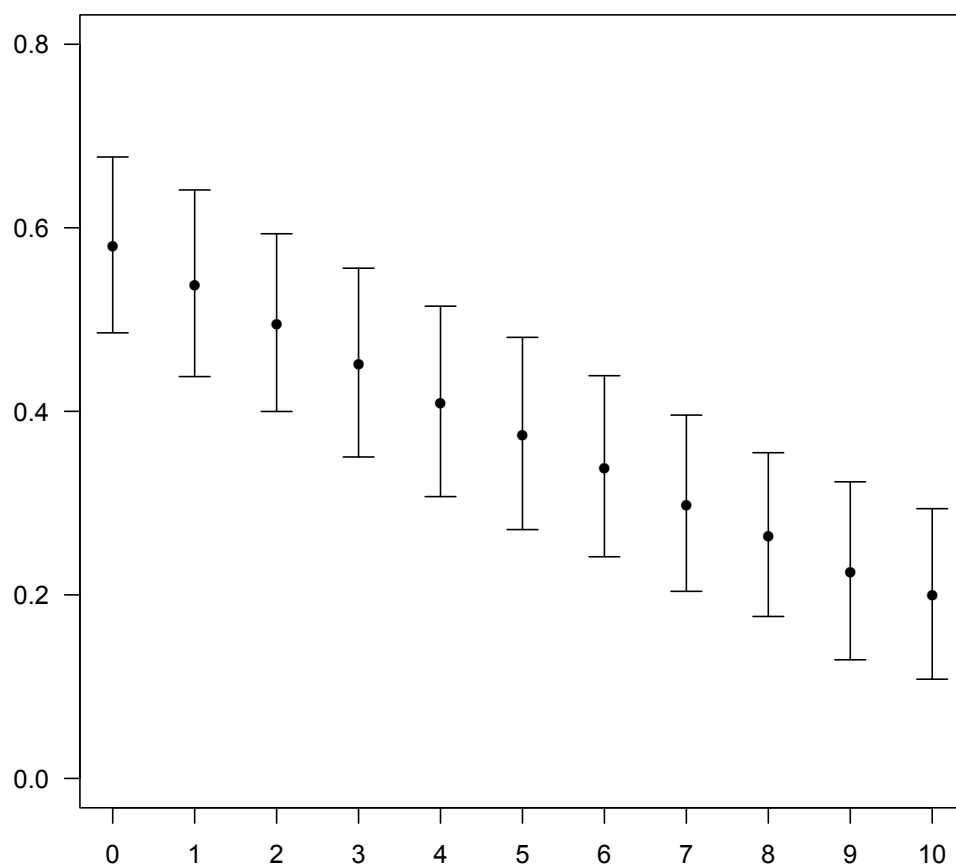

B

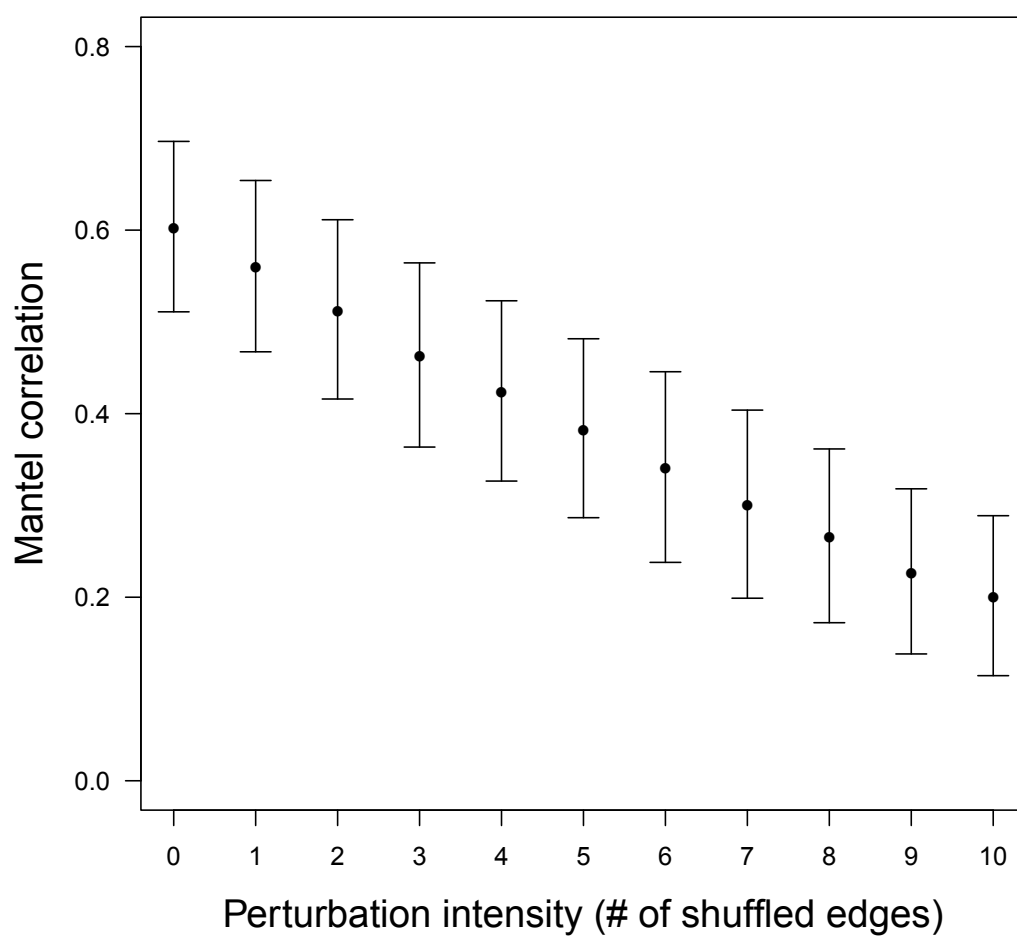

Supplement: S1 Fig — Same legend as in Fig 3 (main text). We used the same simulation settings as in Fig 3 except for the ratio between the HTT rate and the within-genome transposition, which is one per 1000 (panel A) versus one per 100 (this figure, panel B and Fig 3). The result is insensitive to that ratio because (i) the intra-genomic dynamic of TEs is always much faster than their inter-genomic dynamic and (ii) the criterion used to end the simulation remains the number of successful HTTs (here it equals 150). (PDF) [file pbio.2001536.s001.pdf]

**A**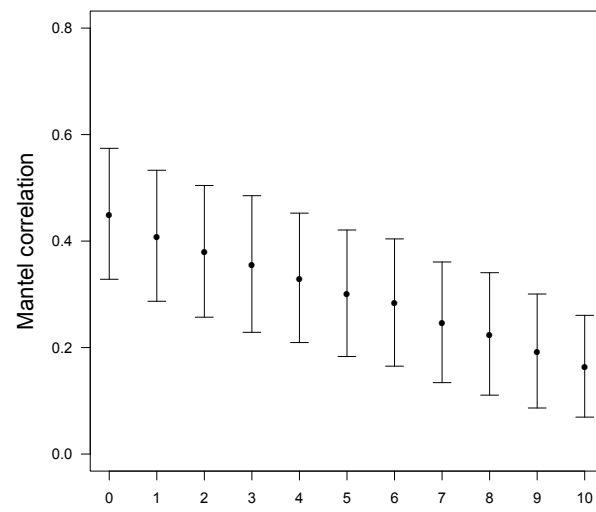**B**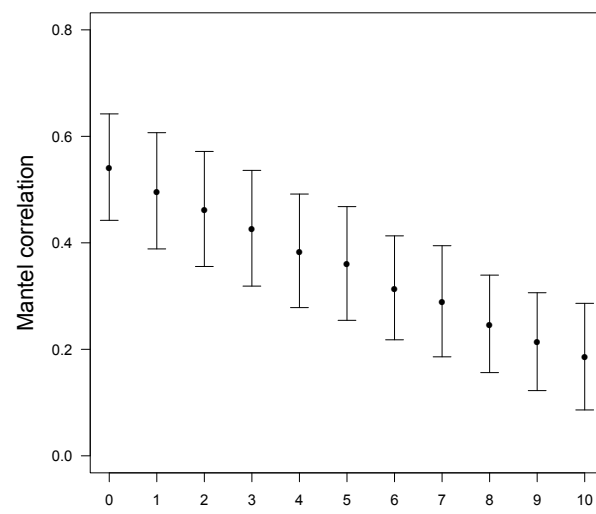**C**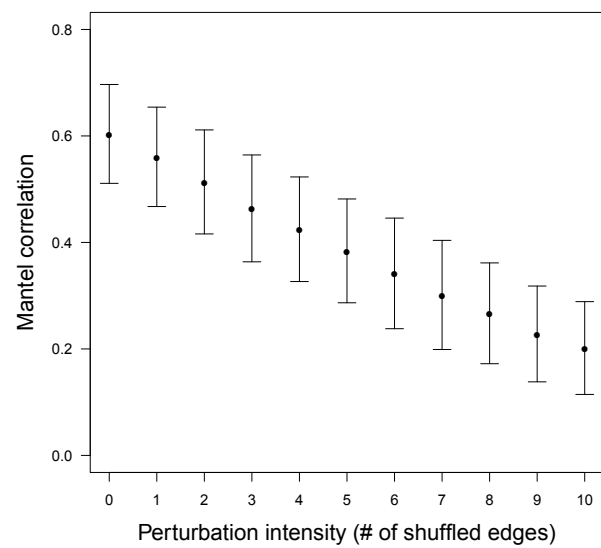

Supplement: S2 Fig — Same legend as in Fig 3 (main text). We used the same simulation settings as in Fig 3. We tested the effect of the number of HTTs on the distribution of Mantel correlation coefficient (n = 50, 100, and 150 for panels A, B, and C, respectively). We show similar trends for all the tested situations (an increasing level of perturbation in network connectivity leads to a decreased correlation between simulated β-matrices). However, the level of correlation increases with the number of successful HTTs within the network. This result means that the network reconstruction will be all the easier when the number of HTTs is high within the species group considered. (PDF) [file pbio.2001536.s002.pdf]
