# Supplementary material for: Ecological networks to unravel the routes to horizontal transposon transfers
Source: PLoS Biol. 2017 Feb 15;15(2):e2001536. doi: 10.1371/journal.pbio.2001536 (PMC5331948; doi:10.1371/journal.pbio.2001536)
Supplement: S3 Fig — Panel A shows different structures of networks in which the nodes (species) have the same average degree of connectivity: random (A1), scale-free (A2), bipartite (A3), and modular (A4) networks (see Box 3 for a detailed description). Panel C represents random networks with different link densities, with the extreme case corresponding to the complete network. Panels B and D describe the diversity of dynamic of propagation of a single TE family in the network. The x-axis represents the number of species infected by the TE. The y-axis corresponds to time (number of iterations) needed to contamination of x species (x-axis) in the network. The box-plots represent the distribution of the time required for the contamination of x species. The width of the box-plot represents the proportion of trajectories in which the TE has contaminated x species. The figure shows networks with distinct topologies (panel A and C) and the dynamics of spread of a unique family of TE in these networks (panel B and D). For each category of networks (panel A and C), 400 simulations were performed. At the beginning of each simulation, a single copy of TE is placed in one of the species of the network, all the 20 network species being tested (20 different initial conditions/modality were therefore tested; 20 repetitions were performed per modality). Panels A and B show that the network structure affects the distribution of the TE propagation dynamics. The scale-free and modular networks generate dynamics different from those obtained in the case of random networks. The scale-free networks generate a very large diversity of TE propagation speeds: TE can spread either very rapidly when hubs are quickly contaminated or very slowly when hubs are slow to be contaminated. In modular networks, trajectories leading to the contamination of a large number of species are rare (strong decrease in the proportion of trajectories with more than 12 contaminated species) because of the presence of partially isola [file pbio.2001536.s003.pdf]

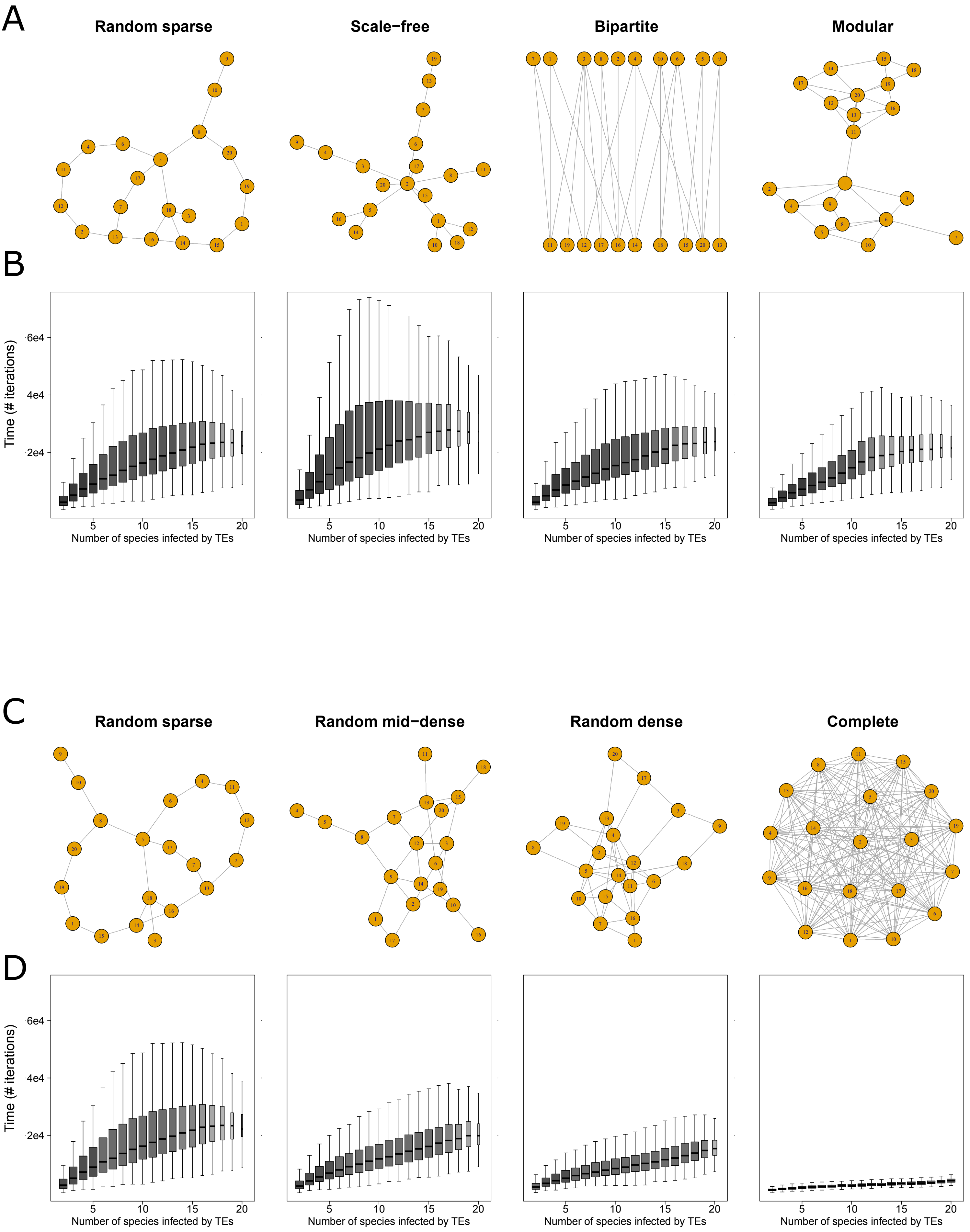
A
Random sparse
Scale−free
Bipartite
Modular
B
Time (# iterations)
6e4
4e4
2e4
5
10
15
20
Number of species infected by TEs
C
Random sparse
Random mid−dense
Random dense
Complete
D
Time (# iterations)
6e4
4e4
2e4
5
10
15
20
Number of species infected by TEs
