# Supplementary material for: Ecological networks to unravel the routes to horizontal transposon transfers
Source: PLoS Biol. 2017 Feb 15;15(2):e2001536. doi: 10.1371/journal.pbio.2001536 (PMC5331948; doi:10.1371/journal.pbio.2001536)
Supplement: S1 Text — (DOCX) [file pbio.2001536.s004.docx]

**S1: Which molecular vehicles for HTT?**

The molecular vehicle function requires organisms having traits that facilitate their entry into the organism and into the cells, taking up and delivering genetic material - including TEs - within the cell. A demographic explosion within the host could be a way to maximize the opportunity to capture or deliver TEs. Their cellular tropism and ability to reach the germline cells would also favor HTTs.

The extent to which organisms may be involved in HTTs as molecular vehicles should strongly depend on five criteria: (i) their host range, (ii) their replication and demography within their host, (iii) their cellular tropism, (iv) their ability to reach the germline cells in recipient species and to access to the nuclear compartment, and (v) their genetic support - DNA or RNA. We detail below that both DNA and RNA viruses can be valuable candidates to be molecular vehicles of TEs. Genetic material derived from all known viral genome types and replication strategies may occasionally enter their host germ line [1–4].

***DNA viruses***: *A priori*, the viral genetic structure that should be most favorable to HTTs would be large DNA genomes containing non-coding regions, which could temporarily carry exogenous genetic material (*e.g.,* the largest known DNA viruses of the Megavirales order with genome size ranging from 100 kb to 1 Mb have evolved by acquisition of cellular sequences including TEs [5]). Furthermore, the synergistic action of DNA viruses (TE molecular vehicles) and their arthropod vectors (ecological connectors) might favor HTTs. For example, the single-stranded DNA plant geminiviruses, which are vectored by whiteflies, leafhoppers and treehoppers, are able to endure experimental insertion and excision of the maize Activator DNA transposon without deleterious effects on their capacity to replicate [6].

***RNA viruses***: The great genetic plasticity and high mutation rates exhibited by RNA viruses allow them to alternate replication in disparate vertebrate and invertebrate hosts, which would favor their implication in HTTs. But, unlike DNA viruses, RNA viruses might impose two major constraints for catalyzing HTTs: first, the integration of TEs in a small viral reverse transcribed genome might be lethal for viruses when viral gene density is high; second, the molecular mechanisms involved in HTT would be rather complex for non-reverse-trancribed RNA viruses, requiring the synthesis of a viral cDNA by an exogenous reverse transcriptase prior to genomic integration. RNA viruses might however promote HTT when TE RNAs are co-packaged along with the viral genomic RNA. This mechanism seems likely because a recent analysis of RNA populations encapsidated by an eukaryotic single-stranded RNA virus (the flock house virus) has revealed that virions frequently package a variety of host-encoded RNAs including those derived from TEs residing in the host genome [7]. Moreover, the viral RNA may recombine with TE RNAs as reported between different viruses co-infecting the same cell, and even occurred between viral and cellular RNAs [7–9]. The expected endogenization process may occur also for non-retroviral RNA viruses [1].

***Exosomes***: Exosomes are small membrane vesicles of endocytic origin secreted by most cell types, and are thought to play important roles in intercellular communications. They contain protein and RNA including miRNA and piRNA and retrotransposon sequences that they can transport from cell to cell [10,11]. TEs packaged in exosomes could therefore be transferred between both somatic and germline cells. Similarly to viruses, TEs could be horizontally transferred between the somatic cells of different organisms, via some kind of vectors (*e.g.,* a macro-parasite).

**References**

1. Katzourakis A, Gifford RJ. Endogenous viral elements in animal genomes. PLoS Genet. 2010;6(11).

2. Gilbert C, Maxfield DG, Goodman SM, Feschotte C. Parallel germline infiltration of a lentivirus in two Malagasy lemurs. PLoS Genet. 2009;5(3):e1000425.

3. Drezen J-M, Gauthier J, Josse T, Bézier A, Herniou E, Huguet E. Foreign DNA acquisition by invertebrate genomes. J Invertebr Pathol. (under press)

4. Hayward A, Katzourakis A. Endogenous retroviruses. Curr Biol.; 2015;25(15):R644–6.

5. Filée J, Chandler M. Gene exchange and the origin of giant viruses. Intervirology. 2009;53(5):354–61.

6. Laufs J, Wirtz U, Kammann M, Matzeit V, Schaefer S, Schell J, et al. Wheat dwarf virus Ac/Ds vectors: expression and excision of transposable elements introduced into various cereals by a viral replicon. Proc Natl Acad Sci U S A. 1990;87(19):7752–6.

7. Routh A, Domitrovic T, Johnson JE. Host RNAs, including transposons, are encapsidated by a eukaryotic single-stranded RNA virus. Proc Natl Acad Sci U S A. 2012;109(6):1907–12.

8. Greene AE, Allison RF. Recombination between viral RNA and transgenic plant transcripts. Science. 1994;263(5152):1423–5.

9. Geuking MB, Weber J, Dewannieux M, Gorelik E, Heidmann T, Hengartner H, et al. Recombination of retrotransposon and exogenous RNA virus results in nonretroviral cDNA integration. Science. 2009;323(5912):393–6.

10. Skog J, Würdinger T, van Rijn S, Meijer DH, Gainche L, Curry WT, et al. Glioblastoma microvesicles transport RNA and proteins that promote tumour growth and provide diagnostic biomarkers. Nat Cell Biol. 2008;10(12):1470–6.

11. Zhou Y, Zheng H, Chen X, Zhang L, Kai W, Guo J, et al. The *Schistosoma japonicum* genome reveals features of host-parasite interplay. Nature. 2009;460(7253):345–51.
