# Supplementary material for: Ecological networks to unravel the routes to horizontal transposon transfers
Source: PLoS Biol. 2017 Feb 15;15(2):e2001536. doi: 10.1371/journal.pbio.2001536 (PMC5331948; doi:10.1371/journal.pbio.2001536)
Supplement: S2 Text — (DOCX) [file pbio.2001536.s005.docx]

**S2: Methods for modeling TE dynamic within the HTT network**

HTT opportunities between two species depend on the probability of each TE copy in the donor genome being transferred, but also on the number of TE copies in the donor genome. The modeling of TE flow requires therefore to take into account both the intra-genomic TE dynamics within each node (amplification dynamics and persistence of TEs within genomes) and the dynamics of transfer along the network links. Our objective is to illustrate the use of network framework in the context of HTT. To that aim, we have developed a simplistic model of the dynamics of TEs, which could however be improved on the basis of different models proposed in the literature (*e.g.,* [1,2]).

The dynamic of each TE family is considered independent of that of other TE families. The method (see below) describes the modeling of a single TE family within the network. Based on computer simulation, our model generates the intra- and inter-genomic (*i.e*., HTT) dynamic of TEs through an iterative process. During each iteration *t* (corresponding to a time step) we calculated for each network node, the number of TE copies in three distinct states: (i) active copies ($n_{a}^{t}$) that have a transposition activity; (ii) inactive copies ($n_{i}^{t}$) that are intact in their sequence but whose transposition activity is blocked by their host species (methylation, etc.); when transferred horizontally those copies can recover their transposition activity in the genome of the recipient species; (iii) degraded copies ($n_{d}^{t})$*,* which have lost their transposition capacity irreversibly (*e.g*., due to a mutation in their sequence), whatever the host genome species.

1. ***Intra-genomic dynamic.*** The modeling of intra-genomic dynamic is based on the number of active, inactive and degraded copies that appear in the genome at iteration *t*, regardless of HTT.

***Inactive copies***: The number of new inactivated copies at iteration *t* ($n_{new.i}^{t}$*)* is determined by sampling active copies at iteration *t*-1 from a binomial distribution:

$n_{new.i}^{t}\sim B(n_{a}^{t-1}{, P}_{inact}(n_{a}^{t-1}+n_{i}^{t-1}))$ (1)

with a probability of inactivation *P_inact_* depending on the number of active and inactive copies of the TE family within the genome. The probability of inactivation is used to control the number of TE copies into the genome, by taking into account the ability of the species to be a TE reservoir. We assume that an intact copy is not initially regulated when arriving into a new genome (the probability of inactivation of an active copy becomes almost null, see Eq. 2 below). The family will invade the genome according to its transposition activity. When a genome capacity reached its limit for that TE family, the probability of inactivation of an active copy will become very high (close to 1, see Eq. 2). The proportion of active copies will become very small relative to the proportion of inactive copies. We assume that the probability of copy inactivation increases with the total number of copies (*k*) of the TE family in the genome, according to a logistic function:

$P_{inact}\left( k \right)=1-1/(1+e^{-0.1\times(n_{\frac{1}{2}}-k)})$ (2)

The parameter $n_{1/2}$ gives the number of copies at the inflection point, which corresponds to the half reservoir capacity of the genome.

The number of active and inactive copies became

$$n_{a}^{t}=n_{a}^{t-1}-n_{new.i}^{t}$$

$$n_{i}^{t}=n_{i}^{t-1}+n_{new.i}^{t}$$

***Active copies*.** The number of new active copies ($n_{new.a}^{t}$*)* at iteration *t* is determined by sampling active copies that remain active at iteration *t*-1 from a binomial distribution:

$n_{new.a}^{t}\sim B(n_{a}^{t},P_{Transp}\times P_{Fix})$ (3)

taking into account the probability of TE transposition ($P_{Transp}$) and of TE fixation ($P_{Fix}$) at a new insertion. The number of active copies became

$$n_{a}^{t}=n_{a}^{t}+n_{new.a}^{t}$$

We consider that an inactivated copy (from an epigenetic inactivation -for example by methylation-) cannot become active again within the same genome as this does not change (or marginally) the intra-genomic dynamics of TEs in our model: the re-activate copy will be inactivated again especially if number of copies of the TE family is large (see eq (2)).

***Degraded copies***. The number of new degraded copies ($n_{new.da}^{t}$) and ($n_{new.di}^{t})$ that appear at iteration *t* from active and inactive copies, respectively, are determined from a binomial distribution:

$n_{new.da}^{t}\sim B(n_{a}^{t},P_{Deg}$) (4)

$n_{new.di}^{t}\sim B(n_{i}^{t},P_{Deg}$) (5)

with a probability of degradation ($P_{Deg}$) considered to be constant and equal for all genome species. The number of active, inactive and degraded copies in the time interval *t* became

$$n_{a}^{t}=n_{a}^{t}-n_{new.da}^{t}$$

$$n_{i}^{t}=n_{i}^{t}-n_{new.di}^{t}$$

$$n_{d}^{t}=n_{d}^{t}+n_{new.da}^{t}+n_{new.di}^{t}$$

In our model, we consider that a degraded copy cannot become active again, although this can occur biologically. It is indeed known that new active TE copies can be formed from the rearrangement of degraded copies; the new type of TE, still uncontrolled by the host, can then invade its genome [3,4]. However, to compare several model outputs, our simulations are made with a constant number of TE types/families, without considering the possible random emergence of new types of TEs (from rearrangement of degraded copies) in the genome of some species of the network.

**II) Inter-genomic dynamic (or HTT dynamic)**

A donor species can transfer TE copies to a recipient species of the network. These copies will be active (capable of transposition in the genome of the recipient species) or degraded (incapable of transposition). The number of copies successfully transferred between two host species is determined in two steps.

1. **Determination of the number of candidate copies for transfer**

The number of active copies ($n_{ac}^{t})$ candidate to the transfer from a donor genome is determined as follows:

$n_{ac}^{t}\sim B((n_{a}^{t}+n_{i}^{t}){, P}_{HTT})$ (6)

from a sampling among the active and inactive copies in the donor species, the inactive copies becoming again active in the genome of the recipient species.

The number of degraded copies ($n_{dc}^{t})$ candidate to the transfer from a donor genome is determined as follows:

$n_{dc}^{t}\sim B((n_{d}^{t}){, P}_{HTT})$ (7)

from a sampling among the degraded copies in the donor species.

1. **Determination of the number of copies successfully transferred**

The effective transfer of TEs depends on the topology of the networks (distribution of connections). In this simplest version, the potential recipient species of a given TE copy is determined from sampling one species within an uniform distribution. The transfer will be effective only if the donor and recipient species are directly connected. Each transferred TE will be fixed in the genome with the probability of fixation ($P_{Fix}$).

For each species, the number of copies successfully received ${(n}_{a.HTT}^{t}$ and $n_{d.HTT}^{t})$ from all other species of the network is determined. The number of active and degraded copies in the time interval *t* became

$$n_{a}^{t}=n_{a}^{t}+n_{a.HTT}^{t}$$

$$n_{d}^{t}=n_{d}^{t}+n_{d.HTT}^{t}$$

**IV) Simulation condition**

In our study, the simulations are based on the dynamics of 30 families of TEs in a network composed of 20 species. In the initial condition (iteration *t*=0), a copy of each TE family is randomly incorporated into one node/species of the network. The dynamic of the TEs continues until the number of 150 successful HTTs is reached, considering all TE families. The probability of transposition ($P_{Transp}$) of a TE copy is equal to 10^-2^, the probability of fixation ($P_{Fix}$) is equal to 0.5, the probability of degradation ($P_{Deg})$ is equal to 10^-4^ and the probability of horizontal transfer ($P_{HTT}$) is equal to 10^-4^. Taking lower probabilities with the same ratio did not change significantly the results but largely increased simulation time (result not shown).

**References**

**Dans la ref Dimitri et al, *Drosophila melanogaster* n'est pas en italiques**

**Dans la ref: Mugnier et al, supprimer SMBE**

1. Le Rouzic A, Boutin TS, Capy P. Long-term evolution of transposable elements. Proc Natl Acad Sci U S A. 2007;104(49):19375–80.

2. Deceliere G, Charles S, Biémont C. The dynamics of transposable elements in structured populations. Genetics. 2005;169(1):467–74.

3. Dimitri P. Constitutive heterochromatin and transposable elements in Drosophila melanogaster. In: Evolution and Impact of Transposable Elements. Springer; 1997. p. 85–93.

4. Mugnier N, Biémont C, Vieira C. New regulatory regions of Drosophila 412 retrotransposable element generated by recombination. Mol Biol Evol. 2005;22(3):747–57.
